# The Arab Region's Contribution to Global Mental Health Research (2009–2018): A Bibliometric Analysis

**DOI:** 10.3389/fpsyt.2020.00182

**Published:** 2020-03-19

**Authors:** Pia Zeinoun, Elie A. Akl, Fadi T. Maalouf, Lokman I. Meho

**Affiliations:** ^1^Department of Psychology, Faculty of Arts and Sciences, American University of Beirut, Beirut, Lebanon; ^2^Department of Psychiatry, Faculty of Medicine, American University of Beirut, Beirut, Lebanon; ^3^Department of Internal Medicine, Faculty of Medicine, American University of Beirut, Beirut, Lebanon; ^4^Department of Health Research Methods, Evidence, and Impact (HE&I), McMaster University, Hamilton, ON, Canada; ^5^University Libraries, American University of Beirut, Beirut, Lebanon; ^6^Department of Political Studies and Public Administration, Faculty of Arts and Sciences, American University of Beirut, Beirut, Lebanon

**Keywords:** mental health, mental disorders, arab countries, Middle East & North Africa (MENA), bibliometrics, citation analysis

## Abstract

**Background:** Mental health research output in the Arab region is increasing, yet little is known about its recent landscape. This study provides a bibliometric analysis of mental health research in all 22 Arab countries over the past decade.

**Method:** We used 760 journals and numerous keywords to search for articles published between 2009 and 2018 by individuals affiliated with institutions located in the Arab region. We analyzed data within Arab countries and between Arab and non-Arab countries.

**Results:** We found that research output in the Arab world has increased by almost 160% in the past ten years, in comparison to 57% for the rest of the world. The quality of publications has also steadily improved, and so did international collaboration. Despite the progress, the number of articles per capita remains remarkably lower for the Arab world compared to the rest of the world. Also, the majority of articles continue to emanate from a limited number of countries (Egypt, Saudi Arabia, and Lebanon) and institutions within these countries. Mental health research topics in the Arab region are similar to those found in low- and middle-income countries of Africa, Asia, Latin America, and the Caribbean.

**Conclusion:** The region needs to invest more in mental health research to close the gap with other medical and healthcare research areas and with the rest of the world. The region also needs to increase its international collaboration and research training to produce higher-quality studies, attract more funding, and publish more in top journals. As the region's population continues to face increasing trauma as a result of war and terrorism, among others, the field is afforded an opportunity to establish a major standing in the healthcare domain. Researchers are uniquely poised to use their body of research evidence to effectively help people reengage with their environments and return to daily life activities.

## Background

Over the last decade, many Arab countries have witnessed wars, conflicts, and major social and geopolitical changes that have negatively influenced the mental health of their people (1, 2). As a result, many researchers examined published literature on the topic, but have focused on specific disorders, such as autism (3), bipolar disorders (4), and substance use/abuse (5). Other studies have focused on specific countries, such as Saudi Arabia (6), the United Arab Emirates (7, 8), and Arabian Gulf countries (9). Still, other studies have focused on a specific scope, such as Muslim mental health (10), or a specific methodology, such as randomized trials (11). In contrast, this study uses bibliometrics (12) to analyze the Arab region's overall contribution to global mental health research from 2009 to 2018, focusing on answering the following research questions:

1. How many journal articles were published during this period in the Arab region and globally?
2. Which countries and institutions in the region contributed substantially to this body of quantitative evidence?
3. In which journals were the articles published?
4. What are the impact and use of these journal articles as measured by citations?
5. To what extent mental health researchers in the Arab region collaborate locally and internationally, and what is the impact of this collaboration as measured by citations?
6. What are the research topics mostly published by local mental health researchers?
7. What challenges do local researchers encounter in carrying out mental health research? What to do about these challenges?

The Arab world consists of 22 countries, including Algeria, Bahrain, Comoros, Djibouti, Egypt, Iraq, Jordan, Kuwait, Lebanon, Libya, Mauritania, Morocco, Oman, Palestine, Qatar, Saudi Arabia, Somalia, Sudan, Syria, Tunisia, United Arab Emirates, and Yemen. From here on, we refer to these countries cumulatively as “Arab countries,” “Arab region,” and “Arab world.” Bibliometric analyses of mental health research in the Arab region are important for several reasons. First, when making funding allocations, agencies consider variables such as research impact, quality, and collaboration potential of academic institutions, which bibliometric analyses provide (13). Second, bibliometric analyses can identify knowledge gaps in important areas of research, which can guide fund allocation. We should emphasize here that while Arab countries spend about 0.5% of the gross domestic product on research, only a fraction of that is spent on non-communicable diseases (NCD), which include mental illness (14). Finally, bibliometric analyses allow academic institutions to compare where they stand in the current research landscape.

The results of this study can be useful in many specialties, including, but not limited to psychiatry, psychology, health sciences, sociology, anthropology, and cultural studies. In addition to helping researchers, clinicians, government officials, and decision-makers understand the overall intellectual structure of the mental health research field in the region, the insights offered by this study are instrumental in ensuring that guidelines, direction, or an action-oriented agenda for future research on mental health are accurately articulated. In the absence of a holistic and comprehensive view of the research field, outstanding issues can remain undetected. This study builds on research carried out by Jaalouk et al. (15) and Karam and Itani (16) who called for more mental health research in the Arab world because of the wide gap in research productivity in this area in comparison to other countries and regions.

## Methodology

### Overall Design

We conducted a comparative analysis of bibliometric indicators within Arab countries and between Arab and other countries. First, we extracted data on Arab countries alone and analyzed nine bibliometric indicators, which we used to describe trends *within* Arab countries (number of articles by country, number of articles relative to country population, number of articles by institution, number of articles by year, research quality, collaboration, research reach, citation count, and research themes). Then, we used the same methodology to compare data *between* Arab countries and the rest of the world for four key indicators (number of journal articles produced by country, per year, published in the top quartile, and produced collaboratively).

### Eligibility Criteria for Journal Articles From the Arab Region

We included articles written in any language, which met the following criteria:

- Country affiliation of authors: We included articles published by individuals affiliated with any of the 22 Arab countries.
- Time-period: We selected the period between 2009 and 2018 to examine recent trends. We conducted the searches on January 1, 2019.
- Types of documents: Unlike Jaalouk et al. and Karam and Itani who included all document types in their studies (i.e., original primary research, reviews, commentaries, letters, case reports, etc.), we included only peer-reviewed journal and review articles, as these are the document types generally accepted as the main instruments for communicating research (17, 18). We excluded commentaries, editorials, abstracts, and other types of documents.

### Search Strategy

#### Database Selection

As described in more detail below, we chose Scopus as the main database and PubMed and Web of Science as supplementary databases for identifying all the articles we analyzed in this study. We chose Scopus as our main database because it covers all documents included in Medline (https://www.scopus.com/), indexes more than 25,000 journals (19, 20), and covers a wide range of local, regional, and international journals, including non-English language journals.

#### Journal Search

Within Scopus, there are 11 subject categories relevant to psychology and psychiatry. The authors reviewed the categories and selected only those that included the terms clinical psychology, psychiatry, and mental health. Therefore, our search targeted all journals indexed in Scopus under the four subject categories of “Psychiatry and Mental Health,” “Psychiatric Mental Health,” “Biological Psychiatry,” and “Clinical Psychology.” This method yielded 710 journals.

To ensure that Scopus did not miss any relevant journals, we used the same strategy in the Web of Science database. Here, we searched for journals under the subjects “Psychiatry” and “Clinical Psychology” and found an additional 50 unique journals, bringing the total number of journals covered in this study to 760—all of which are indexed in Scopus. Scopus indexes these 50 journals under applied psychology, social psychology, general psychology, developmental and educational psychology, and clinical neurology, which explains why we did not detect them in the initial search. Given that the match between the Web of Science database and Scopus was not perfect, we decided to use PubMed to verify whether there are more relevant journals not assigned under any of the subject categories mentioned above but where 75% of the articles had a mental health MeSH term. This search did not result in any additional journals.

#### Keyword Search

To be further inclusive, we additionally searched Scopus for articles that included in their *titles* any of the keywords associated with the MeSH headings “Mental Disorders” and “Mental Health,” which are subsumed under the theme of “Psychiatry and Psychology.” MeSH headings are controlled vocabulary assigned to each document and organized hierarchically by themes. Keywords used include the following MeSH-major headings and all of the subheadings listed under them: anxiety disorders, bipolar and related disorders, disruptive, impulse control, and conduct disorders, dissociative disorders, elimination disorders, feeding and eating disorders, mental health, mood disorders, motor disorders, neurocognitive disorders, neurodevelopmental disorders, neurotic disorders, paraphilic disorders, personality disorders, schizophrenia spectrum and other psychotic disorders, sexual dysfunctions (psychological), sleep-wake disorders, somatoform disorders, substance-related disorders, and trauma and stressor-related disorders. This strategy uniquely identified about 17.5% of the final tally of articles used in this study.

### Indicators

To provide both a broad overview and a detailed analysis of mental health research in the Arab region, we considered the following indicators:

1. Number of articles by country: This indicates the number of peer-reviewed articles produced by a country. We determined the country(ies) for a given article by examining the address(es) of the author(s) in that article. When authors of the same article were from different countries, we gave full credit for each country. We calculated this for Arab countries and other world countries, using the filter by “Country” option in Scopus.
2. Number of articles relative to country population: To understand the output of a country relative to its population size, we divided the total number of articles produced by that country by the number of its inhabitants as reported by the United Nations (2018). We calculated this for all countries.
3. Number of articles by institution: This refers to the number of peer-reviewed articles produced by an institution in the Arab region. We determined the institution(s) for a given article by referring to the institutional affiliation(s) of the author(s) as listed in that study. Again, we gave full credit for each listed institution.
4. Number of articles by year: We assigned a given article to the year during which it was published in the journal. We calculated this for Arab countries individually and for remaining world countries as a whole.
5. Research quality: We examined research quality by calculating the percentage of articles from the Arab region published in journals whose impact factor was ranked in the top quartile.
6. Collaboration: This refers to the extent of scientific collaboration between an Arab country and other countries. We measured it as the number of articles credited to a given country/region with at least one other credited country/region, divided by the country's total number of articles.
7. Research reach: This refers to the extent to which articles from the Arab region are published in international journal outlets, calculated as the number of articles published in non-Arab journals.
8. Number of citations: This indicator is calculated as the number of times an article is cited, according to Scopus. This number serves as a preliminary indicator of the impact of research produced in the Arab region.
9. Research themes: To identify the areas of greatest interest among mental health researchers in the Arab region, we used Elsevier's SciVal to examine all keywords and phrases used within all abstracts and titles of articles published in 2013–2018 (SciVal limits analysis to a maximum of 6 years).

### Data Analysis

The data were exported and analyzed using Microsoft Excel and Access 2016. In the first set of analyses, we used frequency analysis and cross-tabulations to produce figures for all indicators and compared them across the 22 Arab countries. In the second set of analyses, we compared key indicators between Arab countries and other world countries. We also produced a social network analysis showing the patterns of collaboration within Arab countries and between Arab and non-Arab countries.

## Results

### Publications by the Arab World

**Figure 1:**
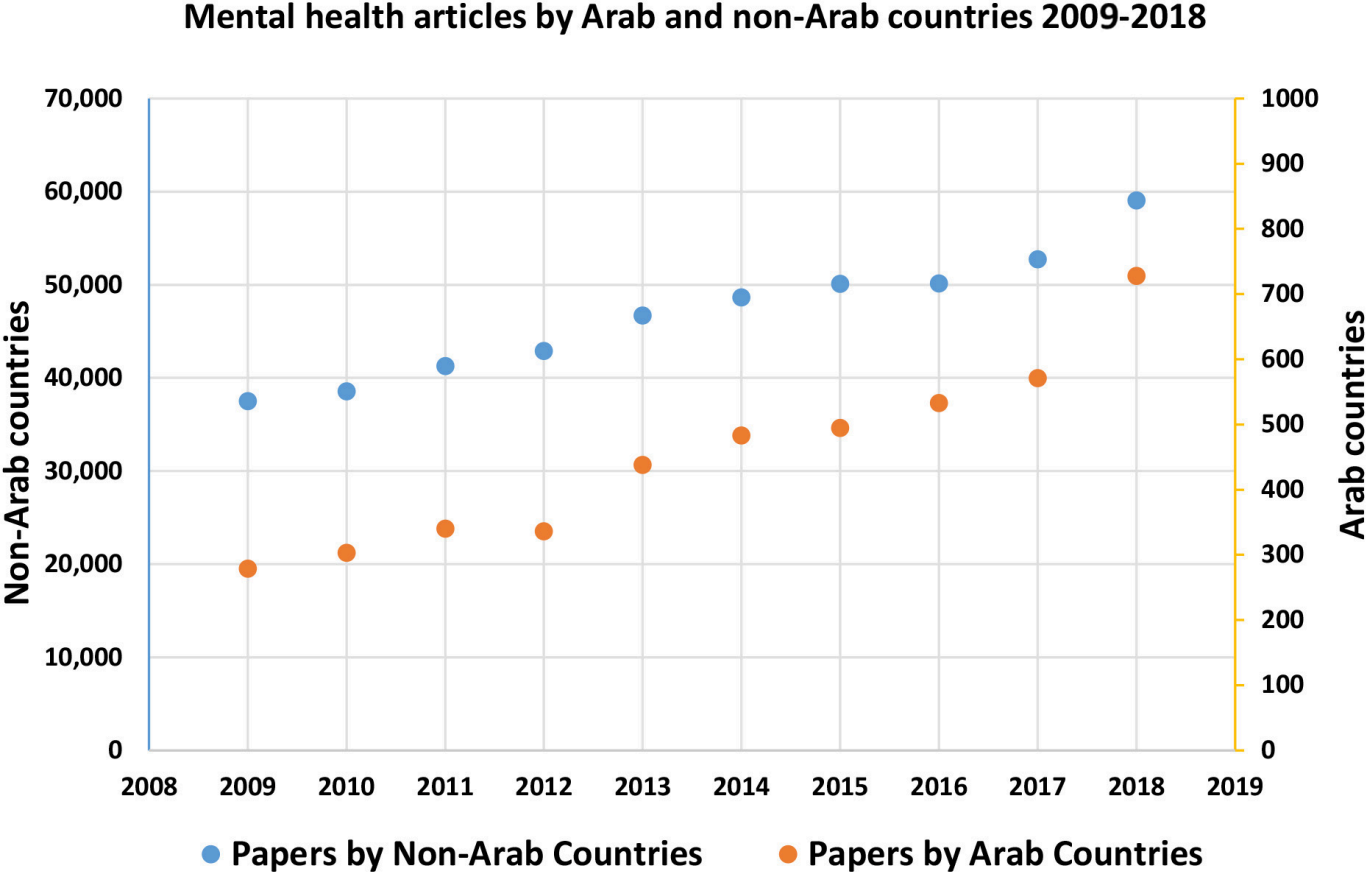
Mental health research published by Arab and non-Arab countries, 2009–2018.

Our search identified a total of 4,506 unique eligible peer-reviewed journal articles published by all 22 Arab countries in the past decade. Considering the 467,533 mental health articles published by non-Arab countries, the Arab region contributed to about 1% of mental health publications globally in 2009–2018. By cross-tabulating the number of articles with the year of publication, we found that the number increased from 279 articles in 2009 to 728 articles in 2018 across the 22 Arab countries. As seen in Figure 1, this reflects a 160% growth in mental health publications, compared to the 57% growth in the rest of the world (37,524–59,038). Nonetheless, despite the accelerated growth, the research output from all Arab countries combined made up only 1.2% of research produced across the globe, in 2018.

### Publications by Arab Countries

**Figure 2:**
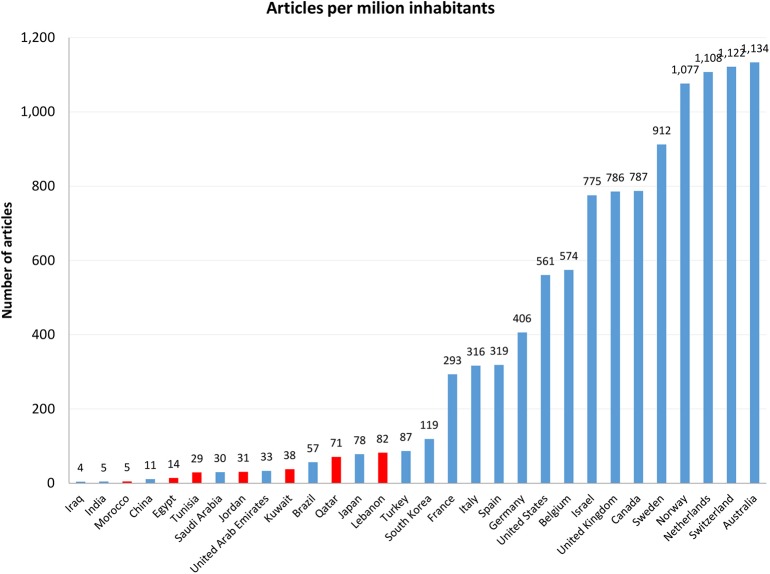
Number of articles per capita, in select Arab and non-Arab countries, 2009–2018.

Table 1 shows the number of articles published by Arab countries. The top five countries in terms of the number of publications were Egypt (1,419), Saudi Arabia (991), Lebanon (502), Tunisia (341), and the United Arab Emirates (319). These countries accounted for about 80% of the region's research output. Table 1 shows the number of publications per capita for Arab countries. Figure 2 shows the number of articles per capita in all Arab countries and the top-ranking non-Arab countries. Lebanon had the highest number of published articles per capita (82), followed by Qatar (71), Kuwait (38), and the United Arab Emirates (33). The great majority of Arab countries lagged behind developed countries.

**Table 1:** Mental health articles published by Arab countries, 2009–2018.

**Rank**	**Country**	**% of Arab population (N = 422,717,409)**	**Number of articles**	**% of Arab region articles**	**Articles per capita**
1	Egypt	23.5%	1,419	31.5%	14
2	Saudi Arabia	7.9%	991	22.0%	30
3	**Lebanon**	1.4%	502	11.1%	**82**
4	Tunisia	2.8%	341	7.6%	29
5	**United Arab Emirates**	2.3%	319	7.1%	**33**
6	**Jordan**	0.6%	304	6.7%	**31**
7	**Qatar**	0.6%	191	4.2%	**71**
8	Morocco	8.6%	176	3.9%	5
9	Iraq	9.3%	172	3.8%	4
10	**Kuwait**	1.0%	159	3.5%	**38**
11	Oman	1.1%	98	2.2%	20
12	Palestine	1.2%	91	2.0%	18
13	**Bahrain**	0.4%	49	1.1%	**31**
14	Algeria	9.9%	39	0.9%	1
15	Sudan	9.8%	36	0.8%	1
16	Syria	4.3%	32	0.7%	2
17	Yemen	6.8%	17	0.4%	1
18	Libya	1.5%	11	0.2%	2
19	Somalia	3.6%	7	0.2%	0
20	Mauritania	1.1%	2	0.0%	0
21	Djibouti	0.2%	1	0.0%	1
22	Comoros	0.2%	0	0.0%	0
	Arab Countries	100.0%	4,506^*a*^	100.0%	11
	Non-Arab Countries	–	467,533	–	61

a *The total number of articles (4,506) does not equal the sum of the column because of collaboration between two or more countries*.

*Countries in bold are top 5 in terms of articles per capita*.

### Publications by Arab Institutions

Table 2 shows the publication characteristics of the top 20 Arab institutions according to the number of articles published from 2009 to 2018. The top five institutions with most publications were also located in Egypt, Saudi Arabia, and Lebanon. In Egypt, Cairo University and Ain Shams University accounted for about 53% of their country's publications, while in Saudi Arabia, King Abdulaziz University and King Saud University produced 51% of their country's publications. In Lebanon, the American University of Beirut produced about 40% of all the country's articles, followed by the University of Balamand group (27%) and Saint Joseph University (20%). In some of the remaining countries, one or two institutions accounted for the majority of the national research output. For example, in Oman, Sultan Qaboos University produced 81% of mental health research while in Kuwait, Kuwait University produced 55% of all research. In Qatar, Weill Cornel Medical College and Hamad Medical Corporation produced 95% of all the country's mental health-related research.

**Table 2:** Publication characteristics of the top 20 Arab institutions according to the number of articles published between 2009 and 2018.

**Rank**	**Institution**	**Articles** **by institution**	**% of national contribution**	**Articles in international journals**	**Articles in top quartile journals**
1	Cairo University (Egypt)	387	27%	108 (28%)	34 (9%)
2	Ain Shams University (Egypt)	367	26%	153 (42%)	47 (13%)
3	King Abdulaziz University (Saudi Arabia)	265	27%	283 (87%)	63 (19%)
4	King Saud University (Saudi Arabia)	239	24%	185 (76%)	37 (15%)
5	American University of Beirut (Lebanon)	199	40%	186 (97%)	57 (30%)
6	Mansoura University (Egypt)	146	10%	72 (48%)	11 (7%)
7	Balamand, IDRAAC, St. George (Lebanon)	138	27%	131 (99%)	103 (78%
8	University of Jordan	126	41%	112 (92%)	19 (16%)
9	Zagazig University (Egypt)	115	8%	52 (44%)	8 (7%)
10	United Arab Emirates University	110	34%	110 (97%)	20 (18%)
11	Razi Hospital (Tunisia)	104	30%	99 (92%)	16 (15%)
12	Université Saint-Joseph (Lebanon)	101	20%	100 (100%)	16 (16%)
13	Assiut University (Egypt)	89	6%	72 (77%)	17 (18%)
14	Hamad Medical Corporation (Qatar)	87	46%	86 (97%)	21 (24%)
15	Kuwait University (Kuwait)	87	55%	81 (92%)	13 (15%)
16	Sultan Qaboos University (Oman)	79	81%	62 (82%)	14 (18%)
17	Alexandria University (Egypt)	71	5%	53 (74%)	12 (17%)
18	Weill Cornell Medical College (Qatar)	71	37%	72 (100%)	15 (21%)
19	Tanta University (Egypt)	67	5%	19 (28%)	4 (6 %)
20	Jordan University of Science and Technology	66	22%	41 (65%)	12 (19%)
	Arab Institutions Total	4,506	-	1,089 (24%)	780 (18%)

### Research Quality

**Figure 3:**
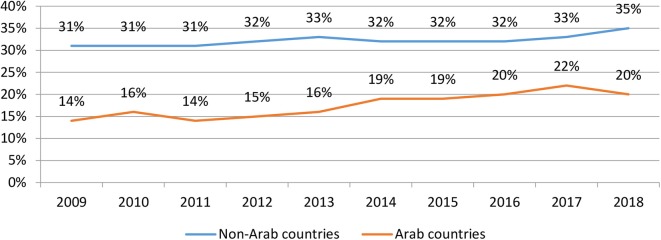
Percent of mental health articles published in top quartile journals by Arab and non-Arab countries.

We examined research quality by calculating the percentage of articles from the Arab region published in journals whose impact factor was ranked in the top quartile. Figure 3 shows that similar to the rest of the world, researchers in the Arab world have been increasingly publishing their work in top quartile journals, improving the ratio from 14% in 2009 to 20% in 2018. This improvement is largely a result of the increase in the percent of articles published in collaboration with researchers overseas, as discussed below.

### Arab and International Collaboration

Unlike collaboration among institutions within the same country, which according to data collected here, remained relatively the same in the past 10 years (ranging from 10 to 12% annually), research collaboration between authors from the Arab region and other countries has increased significantly in the last 10 years. In 2009, the proportion of articles with international collaboration out of all articles published by Arab countries was at 29%, but this number steadily increased to 50% by 2018.

**Figure 4:**
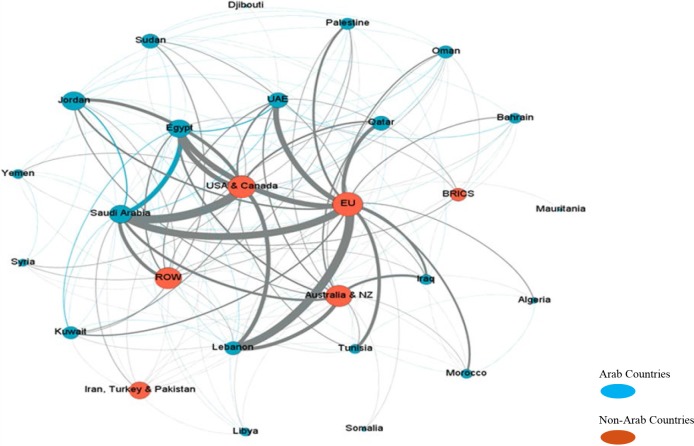
Social network analysis showing the patterns of collaboration among Arab countries and between Arab and non-Arab countries.

Figure 4, generated using the Gephi visualization and exploration software, represents the social network analysis of the collaboration between Arab countries, and between Arab and non-Arab countries. This figure provides only a visual summary; the authors can provide specific metrics upon request. As shown in the figure, collaborations were mostly with the U.S. and Canada (25% of articles), European Union countries (21%), and the United Kingdom (10%). In terms of collaboration within Arab countries, by examining the pattern of collaboration in the top two most published countries (Egypt and Saudi Arabia), it was notable that both countries collaborated more with international researchers (10% of all articles) than with other Arab researchers (5%).

Interestingly, articles without international collaboration showed fewer citations on average (*M* = 3.8) than articles published in collaboration with authors from European countries (*M* = 19.6) and the United States and Canada (*M* = 18.3).

### Rising Trend in Authorship Count

The number of authors per article increased over the years. In 2009, 11.8% of articles were single-authored, and the average number of authors per article was 5.1. However, in 2018, only 5.8% of articles were single-authored, and the average number of authors per article increased to 7.0.

### Publication Outlets

To understand the reach of Arab mental health research, we looked at the journal venues in which investigators in the Arab region have published their work and whether these venues were local or international (Table 3). The three most frequent outlets (as covered by Scopus) were all based in Arab countries and published in Arabic and English - the *Egyptian Journal of Neurology, Psychiatry and Neurosurgery* (469 articles), followed by the Saudi-based *Neurosciences* (278 articles), and the Egyptian *Middle East Current Psychiatry* (259 articles). If Scopus had more comprehensive literature coverage from the Arab world, there might have been more local journals featuring among the most popular outlets for publishing local research on mental health. These results highlight the need for both the establishment of national journals and for having these journals indexed in international databases. These steps will increase the visibility of the Arab countries' work and would most likely help improve the quality as well as the quantity of the published work.

**Table 3:** Journals in which mental health research in Arab countries is frequently published (2009–2018).

**Source Title**	**Journal country**	**Article** **count**	**2018 Impact Factor**	**Quartile**
Egyptian journal of neurology, psychiatry and neurosurgery	Egypt	469	NA	NA
Neurosciences	Saudi Arabia	278	0.892	4
Middle East current psychiatry	Egypt	259	NA	NA
Arab journal of psychiatry	Jordan	200	NA	NA
Egyptian journal of psychiatry	Egypt	250	NA	NA
L'Encéphale: Revue de psychiatrie clinique biologique et thérapeutique	France	102	0.865	4
Neuropsychiatric disease and treatment	New Zealand	65	2.228	3
Epilepsy & Behavior	United States	63	2.368	2, 3^a^
Annales medico-psychologiques	France	51	0.207	4
BMC psychiatry	United Kingdom	49	2.666	2
Journal of affective disorders	Netherlands	46	4.084	1
Neurological sciences	Germany	45	2.484	3
Psychiatry research	Netherlands	37	2.208	2, 3^a^
Perspectives in psychiatric care	United States	35	1.240	3, 4^a^
L'Information psychiatrique	France	31	NA	NA
Addictive disorders & their treatment	United States	31	NA	NA
Asian journal of psychiatry	Netherlands	30	1.932	3
Pharmacology biochemistry and behavior	United States	30	2.773	2, 3^a^
International journal of social psychiatry	United States	30	1.370	3
Issues in mental health nursing	United Kingdom	29	0.977	3, 4^a^

a *The journal is ranked in different quartiles in different subject categories*.

### Research Areas

**Figure 5:**
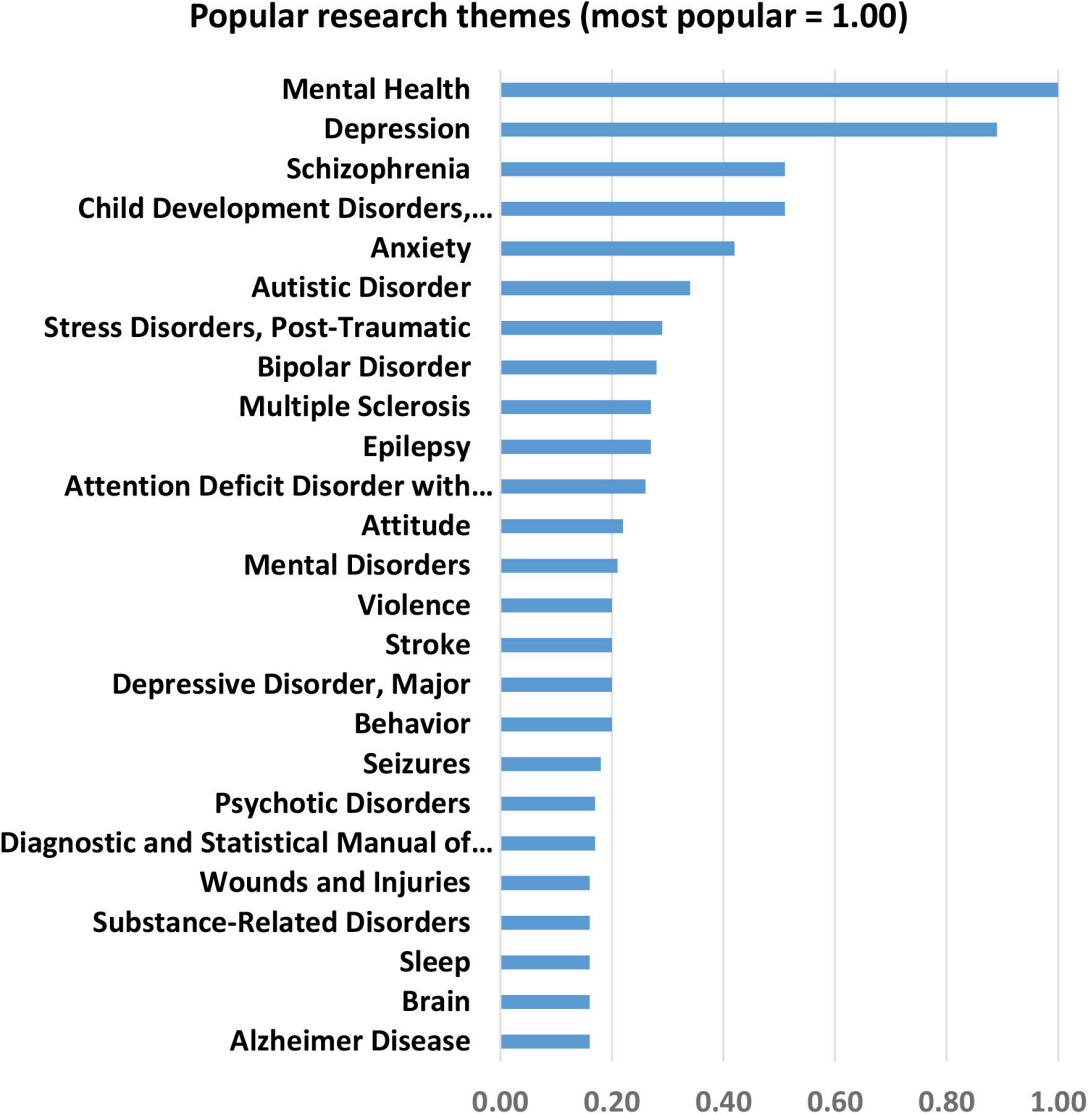
25 Most popular research themes captured by keywords.

A surrogate to important research themes are keywords and phrases that appear most frequently in the title and abstracts fields of examined articles. The frequencies show the popularity of the general term “mental health” as well as depression, schizophrenia, child development disorders, anxiety, Autism, Post-Traumatic Stress Disorder (PTSD), bipolar disorder, and Attention Deficit Hyperactivity Disorder (ADHD), among others, in regional literature (for more, see Figure 5).

## Discussion

This study reports a bibliometric analysis of mental health research in all 22 Arab countries published between 2009 and 2018. We found that research output in the Arab world has increased by almost 160% in the past 10 years, in comparison to 57% for the rest of the world. The quality of publications has also steadily improved, where the proportion of articles published in top quartile journals increased from 14% in 2009 to 20% in 2018. In a similar trend, researchers have become more collaborative, as evidenced by the increase in the number of authors per article. We additionally found that articles with international collaborators receive more citations than those without international collaborators. Despite much progress, the number of articles per capita remains remarkably lower for the Arab world compared to the rest of the world. Also, the majority of articles emanate from a limited number of countries (i.e., Egypt, Saudi Arabia, and Lebanon) and institutions within these countries.

### Key Trends in the Arab Region

The increase in mental health research output in the Arab region is clear. Although it is difficult to contrast our results with other bibliometric studies because of different methodologies, it is possible to draw some tentative comparisons. For instance, Jaalouk et al. (15) found that the Arab region published 1,114 articles related to mental health between 1999 and 2006. Compared to our data, this suggests an ~3-fold increase in the past decade, compared to 2.2 fold for the world as a whole.

Interestingly, the increase in publications over the last decade is driven by some countries more than others. For example, according to data from Jaalouk et al. (15), the United Arab Emirates had no articles before 1985 and about 12 publications per year between 1996 and 2005. However, the United Arab Emirates now accounts for 7% of all publications from the region and ranks fourth regionally in terms of articles published per million inhabitants. Conversely, in 2006, Kuwait was among the top four Arab countries in mental health research output (15) but now ranks 10th regionally in terms of the total number of publications. Other countries like Lebanon, Egypt, and Saudi Arabia have maintained a steady flow of publications over the last decade.

What seems to have changed significantly over the years is the landscape of journal outlets. Afifi (21) found that Arab mental health articles indexed in PubMed between 1987 and 2002 were mostly published in international journals that had impact factors rated in the 1st or 2nd quartiles (e.g., Psychological Reports, Acta Psychiatrica Scandinavica). In the current study, however, it is notable that the most frequent publication outlets during the 2009–2018 period were local journals from Egypt and Saudi Arabia. These journals have impact factors in the 4th quartile or are not rated. The reasons for this shift may have to do with increased coverage of local and regional journals in international databases, increased Arabization and empowerment of local journals, increased competition for publishing in international journals, or researchers finding it more meaningful to publish in local journals. We believe more authors would target local scientific journals (which are typically owned by national universities) if these journals increase their presence or coverage in international databases, such as PubMed, Scopus, and Web of Science.

It is interesting to compare results not only to previous data from the Arab region, but also to current global trends in mental health publications, and to Arab trends in comparable areas of research. In terms of productivity, the Arab region contributes only 1.2% of global mental health research, despite comprising almost 6% of the world's population. Compared to overall medical research in the region, mental health research is lagging. While we found that mental health research increased from 0.7% of global output in 2009 to 1.2% in 2018, El Rassi et al. (22) reported that overall medical research output in the same Arab region increased from 1.0% in 2007 to 2.1% of global output in 2016. Regional mental health research and overall medical research in general, however, share other characteristics: most of the research is published in low-impact local journals (ranging from 20 to 24% of total output) and about half of the studies are collaborative, most frequently with the same non-Arab countries (22).

Finally, there are noted similarities between the Arab countries and non-Arab countries. Particularly, Arab countries, like many others, have become increasingly collaborative in research. For example, while international collaboration in general increased from 3 to 20% between 1980 and 2008 (23), collaboration between Arab countries and European countries and the USA also increased from 39% in 2009 to 52% in 2018. At a more granular level, we note that Egypt and Saudi Arabia (the most populous countries, and the region's largest research producers), collaborated more with international researchers than with Arab researchers; this is in contrast to what we typically see in large non-Arab countries, which tend to have *more* local collaboration because there are many collaborators within their national boundaries (23). We may explain this trend by the fact that despite the size of Egypt and Saudi Arabia, the number of institutions and, by extension, the number of research groups within these two countries, continues to be limited, and researchers may need to look beyond their national borders for collaborators. It is also likely that local authors tend to collaborate with researchers overseas because it reduces the challenge of writing manuscripts in a foreign (typically English or French) language, and facilitates publication in high-ranked journals. Finally, researchers might find that international collaboration allows them to build more sustainable partnerships that open doors for international funding, and allow research to progress faster, in an era of increased pressure to publish.

In terms of commonly researched topics, there is a continued interest in depression, and anxiety, consistent with earlier findings (21), and a possible increase in childhood-related disorders. This trend may reflect research interests, clinical subspecialties, funding priorities, and convenient access to specific clinical populations.

## Strengths and Limitations

Unlike previous bibliometric studies on mental health in the Arab region, this is the only study that looked at indicators of collaboration and quality of research. Additionally, its comprehensive search strategy and use of MeSH headings captured additional articles published in multidisciplinary journals. One possible limitation of this study, however, may have been its selection of four out of 11 possible journal subject categories within the Scopus database. This selection excluded categories like *Developmental and Educational Psychology* and *Neuropsychology and Physiological Psychology*, which may have included entries relevant to mental health. However, when designing the methodology of this study, we informally tested whether these categories would have added incremental value to the data, and we found that they yielded many false-positive articles that were not relevant to mental health. Another limitation of this study is that it included articles from journals covered in Scopus only. Many researchers in the Arab world publish their work in local or national journals not covered in Scopus. Future studies should consider covering such databases as *Al-Manhal, Dar Almandumah*, and *Iraqi Academic Scientific Journals*, which index hundreds of journals published in Arab countries.

## Conclusion and Implications

In the past 50 years, mental health research in the Arab region has been steadily on the rise (15, 21). Unlike previous studies, here we focused on the quantity, quality, and other aspects of this research, covering the period 2009–2018. Our findings suggest that Arab mental health researchers and institutions have been invested in enhancing their research quality and quantity. Indeed, to continue this growth, and make an evidence-informed call for action, authors on this manuscript and other stakeholders, including Arab mental health researchers, institutional and funding agency officials, journal editors and international research collaborators, convened during the 49th Middle East Medical Assembly held at the American University of Beirut in April 2018. They discussed the current status of mental health research in the Arab region, the challenges to its enhancement, and subsequently proposed an action plan (24). The challenges identified included prevalent stigma and low awareness of mental health needs and services, conflict, and war that impede research, scarce institutional and funding resources, inadequate publishing opportunities, insufficient training in mental health research, and shortage of reliable and valid assessment tools. The authors proposed methods for addressing stigma and spreading awareness, increasing collaborative efforts, building research infrastructure, strengthening the mental health workforce, and translating research findings into a call to action on societal and governmental levels (24).

These issues are congruent with the implications of this study, which highlight the gap between the current research output and the documented need for mental health studies in the region. Researchers need to design better studies that are locally relevant yet also contribute to the international literature and therefore have higher chances of being funded and published in highly visible journals. At the level of institutional support and policy, Arab researchers would also benefit from additional funding that targets under-researched themes, and encourages cross-Arab collaboration. Technical support in the form of advanced research training, and copyediting in English, can also improve the chances of producing studies of publishable quality. Although the process may be more complex than stated, we hope that Arab mental health researchers would build on such initiatives to further develop their research portfolios in a way that is impactful locally, regionally, and internationally (25–28). As the region's population continues to face increasing trauma as a result of war and terrorism, among others, the field is afforded an opportunity to establish a major standing in the healthcare domain. Researchers are uniquely poised to use their body of research evidence to effectively help people reengage with their environments and return to daily life activities.

## Data Availability Statement

The raw data supporting the conclusions of this article will be made available by the authors, without undue reservation, to any qualified researcher.

## Author Contributions

All authors contributed to the conception and design of the work as well as the acquisition, analysis and interpretation of the data. All authors also contributed to drafting the work and revising it critically for important intellectual content.

### Conflict of Interest

The authors declare that the research was conducted in the absence of any commercial or financial relationships that could be construed as a potential conflict of interest.

## References

[1] Hassan G Ventevogel P Jefee-Bahloul H Barkil-Oteo A Kirmayer LJ. Mental health and psychosocial wellbeing of Syrians affected by armed conflict. Epidemiol Psychiatr Sci. (2016) 25:129–41. 10.1017/S2045796016000044 26829998 PMC6998596

[2] Maalouf FT Ghandour LA Halabi F Zeinoun P Shehab AA Tavitian L. Psychiatric disorders among adolescents from Lebanon: prevalence, correlates, and treatment gap. Soc Psychiatr Epidemiol. (2016) 51:1105–16. 10.1007/s00127-016-1241-4 27246607

[3] Alnemary FM Alnemary FM Alamri YA Autism research: where does the arab world stand? Rev J Autism Dev Disord. (2017) 4:157–64. 10.1007/s40489-017-0104-6

[4] Kronfol Z Zakaria Khalil M Kumar P Suhre K Karam E McInnis M. Bipolar disorders in the Arab world: a critical review. Annals N Y Acad Sci. (2015) 1345:59–66. 10.1111/nyas.12652 25656934

[5] Sweileh WM Zyoud SH Al-Jabi SW Sawalha AF. Substance use disorders in Arab countries: research activity and bibliometric analysis. Substance Abuse Treat Preven Policy. (2014) 9:33. 10.1186/1747-597X-9-33 25148888 PMC4144697

[6] Koenig HG Al Zaben F Sehlo MG Khalifa DA Al Ahwal MS. Current state of psychiatry in Saudi Arabia. Int J Psychiatr Med. (2013) 46:223–42. 10.2190/PM.46.3.a 24741832

[7] Neves K Lammers WJ. Growth in biomedical publications and scientific institutions in the Emirates (1998–2004): an Arabian renaissance? Health Inform Libraries J. (2007) 24:41–9. 10.1111/j.1471-1842.2007.00699.x 17331143

[8] Osman OT Afifi M. Troubled minds in the Gulf: mental health research in the United Arab Emirates (1989–2008). Asia Pacific J Public Health. (2010) 22(3_Suppl):48S−53S. 10.1177/1010539510373025 20566533

[9] Hickey JE Pryjmachuk S Waterman H. Mental illness research in the Gulf Cooperation Council: a scoping review. Health Res Policy Syst. (2016) 14:59. 10.1186/s12961-016-0123-2 27492156 PMC4972953

[10] Altalib HH Elzamzamy K Fattah M Ali SS Awaad R. Mapping global Muslim mental health research: analysis of trends in the English literature from 2000 to 2015. Global Mental Health. (2019) 6:e6. 10.1017/gmh.2019.3 31157114 PMC6533849

[11] Takriti Y El-Sayeh HG Adams CE. Internet-based search of randomised trials relevant to mental health originating in the Arab world. BMC Psychiatr. (2005) 5:30. 10.1186/1471-244X-5-30 16045805 PMC1199527

[12] De Bellis N Bibliometrics and Citation Analysis: From the Science Citation Index to Cybermetrics. Lanham, MD: Scarecrow Press (2009).

[13] Pincus HA Henderson B Blackwood D Dial T. Trends in research in two general psychiatric journals in 1969-1990: research on research. Am J Psychiatr. (1993) 150:135–42. 10.1176/ajp.150.1.135 8417556

[14] Regan M Gater R Rahman A Patel V. Mental health research: developing priorities and promoting its utilization to inform policies and services. EMHJ-Eastern Mediterr Health J. (2015) 21:517–21. 10.26719/2015.21.7.517 26442893

[15] Jaalouk D Okasha A Salamoun MM Karam EG. Mental health research in the Arab world. Soc Psychiatr Epidemiol. (2012) 47:1727–31. 10.1007/s00127-012-0487-8 22388974

[16] Zarrouq B Bendaou B Elkinany S Rammouz I Aalouane R Lyoussi B. Mental health research in the Arab world: an update. BJPsych Int. (2015) 12:S-25–8. 10.1192/S2056474000000829

[17] Carpenter M Narin F The subject composition of the world's scientific journals. Scientometrics. (1980) 2:53–63. 10.1007/BF02016599

[18] Moed HF Differences in the construction of SCI based bibliometric indicators among various producers: a first over view. Scientometrics. (1996) 35:177–91. 10.1007/BF02018476

[19] Sweileh WM. Bibliometric analysis of medicine–related publications on refugees, asylum-seekers, and internally displaced people: 2000–2015. BMC Int Health Human Rights. (2017) 17:7. 10.1186/s12914-017-0116-4 28320410 PMC5360014

[20] Falagas ME Pitsouni EI Malietzis GA Pappas G. Comparison of PubMed, scopus, web of science, and Google scholar: strengths and weaknesses. FASEB J. (2008) 22:338–42. 10.1096/fj.07-9492LSF 17884971

[21] Afifi MM. Mental health publications from the Arab world cited in PubMed, 1987–2002. Eastern Mediterr Health J. (2005) 11:319–28. 16602450

[22] El Rassi R Meho LI Nahlawi A Salameh JS Bazarbachi A Akl EA. Medical research productivity in the Arab countries: 2007-2016 bibliometric analysis. J Global Health. (2018) 8:020411. 10.7189/jogh.08.020411 30410737 PMC6220353

[23] Larivière V Grant J. Bibliometric analysis of mental health research: 1980–2008. Rand Health Quart. (2017) 6:12. 28845350 10.7249/rhq-06-02-12 PMC5568166

[24] Maalouf FT Alamiri B Atweh S Becker AE Cheour M Darwish H.. Mental health research in the Arab region: challenges and call for action. Lancet Psychiatr. (2019) 6:961–6. 10.1016/S2215-0366(19)30124-5 31327707

[25] Sharan P Gallo C Gureje O Lamberte E Mari JJ Mazzotti G. Mental health research priorities in low- and middle-income countries of Africa, Asia, Latin America and the Caribbean. Br J Psychiatr. (2009) 195:354–63. 10.1192/bjp.bp.108.050187 19794206 PMC3432479

[26] Collins PY Patel V Joestl SS March D Insel TR Daar AS.. Grand challenges in global mental health. Nature. (2011) 475:27–30. 10.1038/475027a 21734685 PMC3173804

[27] da Silva ATC Hanlon C Susser E Rojas G Claro HG Quayle J.. Enhancing mental health research capacity: emerging voices from the National Institute of Mental Health (NIMH) global hubs. Int J Mental Health Syst. (2019) 13:21. 10.1186/s13033-019-0276-9 30988696 PMC6446384

[28] Thornicroft G Cooper S Bortel TV Kakuma R Lund C. Capacity building in global mental health research. Harvard Rev Psychiatr. (2012) 20:13–24. 10.3109/10673229.2012.649117 22335179 PMC3335140

